# Pragmatically Framed Cross-Situational Noun Learning Using Computational Reinforcement Models

**DOI:** 10.3389/fpsyg.2018.00005

**Published:** 2018-01-30

**Authors:** Shamima Najnin, Bonny Banerjee

**Affiliations:** ^1^Department of Electrical and Computer Engineering, University of Memphis, Memphis, TN, United States; ^2^Institute for Intelligent Systems, University of Memphis, Memphis, TN, United States

**Keywords:** cross-situational learning, deep reinforcement learning, Q-learning, neural network, joint attention, prosodic cue

## Abstract

Cross-situational learning and social pragmatic theories are prominent mechanisms for learning word meanings (i.e., word-object pairs). In this paper, the role of reinforcement is investigated for early word-learning by an artificial agent. When exposed to a group of speakers, the agent comes to understand an initial set of vocabulary items belonging to the language used by the group. Both cross-situational learning and social pragmatic theory are taken into account. As social cues, joint attention and prosodic cues in caregiver's speech are considered. During agent-caregiver interaction, the agent selects a word from the caregiver's utterance and learns the relations between that word and the objects in its visual environment. The “novel words to novel objects” language-specific constraint is assumed for computing rewards. The models are learned by maximizing the expected reward using reinforcement learning algorithms [i.e., table-based algorithms: Q-learning, SARSA, SARSA-λ, and neural network-based algorithms: Q-learning for neural network (Q-NN), neural-fitted Q-network (NFQ), and deep Q-network (DQN)]. Neural network-based reinforcement learning models are chosen over table-based models for better generalization and quicker convergence. Simulations are carried out using mother-infant interaction CHILDES dataset for learning word-object pairings. Reinforcement is modeled in two cross-situational learning cases: (1) with joint attention (Attentional models), and (2) with joint attention and prosodic cues (Attentional-prosodic models). Attentional-prosodic models manifest superior performance to Attentional ones for the task of word-learning. The Attentional-prosodic DQN outperforms existing word-learning models for the same task.

## 1. Introduction

Infants face many complex learning problems, one of the most challenging of which is learning a language. It is nothing short of a scientific miracle how quickly and effortlessly they learn a language. The process of language acquisition is multisensory that involves hearing utterances, seeing objects in the environment, and touching and pointing toward them. The ability to map words onto concepts/referents/objects is at the core of language acquisition. The mapping between words and their referents is fundamentally ambiguous as illustrated by Quine using the “Gavagai” problem (Quine et al., 2013). In this problem, the word “Gavagai” is uttered while pointing toward a rabbit in a field; therefore, corresponding referents can be the rabbit, the field or the color of the rabbit. Solving this problem involves a number of challenging tasks: segmenting continuous speech into words, determining a set of objects/referents/concepts that are present in the immediate environment, and finding a way to correlate the heard words with the seen objects. In reality, solving the problem becomes harder because: (1) the infants hear continuous speech consisting of a train of words instead of an isolated word, (2) an object might be mentioned that is absent in the immediate environment, (3) not all words refer to objects, such as verbs and function words, and (4) the referent object is not always touched or pointed to. Thus, there are multiple possibilities in both spaces, language and referent, and learning the mapping between them is a non-trivial problem.

One of the prominent solutions to this problem is cross-situational learning (Gleitman, 1990; Plunkett, 1997; Akhtar and Montague, 1999; Bloom, 2000; Saffran, 2003; Smith et al., 2011; Pinker, 2013) which hypothesizes that co-occurrences of spoken words and their possible referents/objects/events help the infants to learn word meanings across multiple communicative contexts. Each individual interactive situation may be referentially ambiguous. Co-occurrence statistics over many such situations gradually resolve the ambiguity (Räsänen and Rasilo, 2015). Developmental researchers claim that social-interaction cues play a major role in guiding infants' learning and in developing the link between words and objects in the world (Baldwin, 1993; Tomasello and Akhtar, 1995). It is suggested that attention on objects does not necessarily establish the word-referent link unless it is within a task to achieve a goal (Rohlfing et al., 2016). When both the child and caregiver, involved in an interaction, attend to an event to accomplish a goal (such as to learn the name of an object), the situation is called *pragmatically-framed*. The social-pragmatic account of language acquisition hypothesizes that social-cognitive skill (i.e., the ability to infer the speaker's intention during an interaction) is the key to language acquisition (Baldwin, 1993; Bloom, 2000; Bowerman and Levinson, 2001). In support of this theory, it has been shown that 9–10 month old infants failed to learn phonetics from digital videos due to the absence of a live person who could provide social cues and referential information (Kuhl et al., 2003). Behavioral experiments on 18 month old show that they track the speaker's attention and infer the speaker's intention to determine the novel word-referent pairs (Baldwin, 1993; Bowerman and Levinson, 2001).

### 1.1. Review of computational models of word-learning

**Figure 1:**
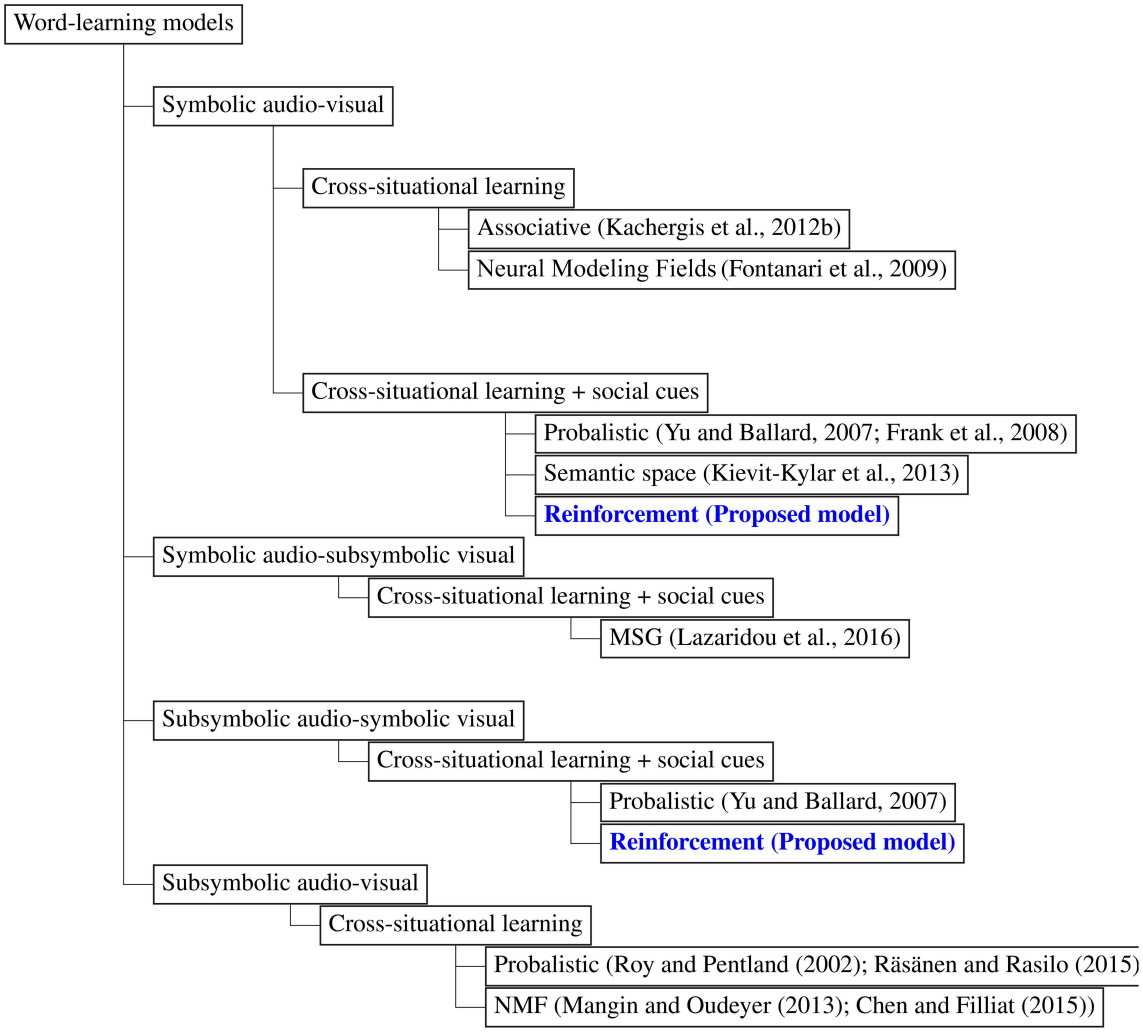
A taxonomy tree of existing word-learning models, including the models proposed in this paper.

Computational modeling of word-learning has been a powerful tool for unraveling the underlying factors and mechanisms of word-learning in infants. It helps to examine psycholinguistic theories of word-learning. In developmental robotics, it plays an important role in the design of a robot's behavioral and cognitive capabilities (Lungarella et al., 2003; Cangelosi and Riga, 2006). Several computational models of word-learning have been proposed, a taxonomy tree of which is shown in Figure 1.

One of the first models for learning word meanings is rule-based (Siskind, 1996). It is capable of learning word meanings from multiple words and multiple meanings in ambiguous context, as in the real world. A synthetic dataset of utterances paired with conceptual expressions are generated where the concepts are considered as utterance meanings. In this model, synthetic representation of utterances and meanings does not fit the distributional properties of the input received by a child. This model does not revise the meaning of a word once it is learned which makes the model incapable of handling highly noisy or ambiguous data.

In Yu and Ballard (2007), a unified model is proposed for learning words-referent pairs by integrating cross-situational evidence and social cues. The speaker's visual attention and prosodic cues in infant-directed speech are considered as social cues. The input data (or observation) consists of utterances (mother's speech) represented as bags of words and meanings represented by manually identifying objects present in the immediate environment. The model uses expectation maximization (EM) to learn correct mappings. Four models are trained: (1) the baseline model using the cross-situational information, (2) the model integrating visual attentional cues with statistical information, (3) the model integrating prosodic cues with statistical information, and (4) the model integrating both kinds of social cues with statistical information. It is found that the model using both visual attention and prosodic cues outperforms the other three models. But it lacks cognitive plausibility because of its non-incremental and intensive batch-processing learning procedure.

In Frank et al. (2008), a Bayesian framework for modeling word-learning is proposed. Using the mother-infant interaction CHILDES dataset the speaker's intent is modeled as a subset of the objects observed when an utterance is formed. Certain properties of word-learning in children, such as the mutual exclusivity bias and fast mapping, are observed in the model. The choice of prior enforces smaller lexicon learning; learning all existing word-object pairs is not a priority. Equally-likely assumption on all intentions indicates that the framework is not incorporating a fully elaborate model of the speaker's communicative intentions.

An incremental, associative model that learns words from context is proposed in Kachergis et al. (2012a). The association score of a word-object pair increases if the pair has co-occurred before or if the referent object is not associated with other words. Empirical evaluations show that associative models match human behavior more closely (Kachergis et al., 2012b; Kachergis and Yu, 2014). One of the drawbacks of the associative model in Kachergis et al. (2012a) is that it uses several parameters with different values without explaining the intuition behind them.

In Kievit-Kylar et al. (2013), a combination of semantic space models is used to learn word-concept pairings. A generalized technique transforms a semantic space model into a word-concept learning model. There are two phases: learning and prediction. In the learning phase, the model is applied to utterances consisting of words. The word tokens and concept tokens are concatenated into a single concept sensory episode. In the prediction phase, an object/concept token is assigned to each word token. Hybrid models that combine some of the existing semantic models outperform each of them. In this model, no social cues are considered.

In Lazaridou et al. (2016), a distributed word-learning model is proposed that works with realistic visual scenes. Distributed linguistic and visual information is used with Multimodal Skip-Gram (MSG) model (Lazaridou et al., 2015) for cross-situational learning. Social cues, such as eye gaze, gestures, and body posture, are used in multimodal learning. Besides learning word-object mapping, the model simultaneously learns word representations that help to infer the appearance of the objects, group concepts into categories, and represent both abstract and concrete words in the same space. Their visual perception is sensory while the audio perception is symbolic. Thus, it ignores the effect of non-linguistic aspects of the caregiver's speech.

In Fontanari et al. (2009), biologically-plausible Neural Modeling Fields is proposed to solve the word-learning problem on a synthetic dataset. The original problem is that of online learning which is relaxed to a batch learning problem. Incorrect associations are removed automatically by a clutter-detection model. The two mechanisms, batch learning and clutter detection, allow the neural modeling fields to infer correct word-object pairings. However, the model assumes that the speech is segmented and the words are represented symbolically.

The continuous nature of speech and visual data are taken into account in Roy and Pentland (2002) and Räsänen and Rasilo (2015). The CELL model (Roy and Pentland, 2002) considers the shape of single objects as the visual input which bypasses referential uncertainty. In Roy and Pentland (2002) and Räsänen and Rasilo (2015), the contextual noise and ambiguity in visual data is considerably less than that experienced by children. CELL was later followed by the model of Yu and Ballard (2004). Phoneme sequences co-occurring with the same objects are grouped together. The common structure of these phoneme sequences across multiple occurrences of the same context are taken as word candidates. Expectation-maximization is used to learn word meanings. It is found that word segmentation can be facilitated by analyzing the acoustic input across communicative contexts instead of modeling speech patterns in isolation (Roy and Pentland, 2002; Yu and Ballard, 2004). However, to make the learning problem simpler, the speech is converted into phoneme-like sequences using pre-trained neural network classifiers before further processing. Though the model uses realistic representation of audio and visual data, none of them consider social cues.

In Mangin and Oudeyer (2013), it is hypothesized that word meanings are learned through multimodal correlation. For finding multimodal association, two types of dataset are used: motion and audio. A non-negative matrix factorization (NMF)-based multimodal unsupervised learning approach is proposed that is able to discover elementary gestures performed by a human and their names in subsymbolic audio and motion streams. In Chen and Filliat (2015), NMF is utilized to learn noun and adjective in cross-situational scenario. In this model, a statistical filtering is used to remove noise from speech, a phoneme recognizer is used to convert continuous speech to symbolic representation, and finally NMF is used to discover word meanings in the visual domain.

The model in Frank et al. (2008, 2009) exhibits language-learning phenomena with mutual exclusivity and fast-mapping as consequences of the structure of the model. Kachergis et al. (2012a) considers mutual exclusivity as a built-in bias in their single-hypothesis model. An associative memory noisily stores everything the model sees. Alishahi (2010) observes, some other associative models (Plunkett et al., 1992; Schafer and Mareschal, 2001; Regier, 2005) show a pattern of vocabulary spurt, similar to that observed in children, where the input data consists of distributed representation of both words and referents. Alishahi (2010) further observes that the incremental clustering-based associative model in (Li et al., 2004, 2007) simulate vocabulary spurt. Other competition-based models (MacWhinney, 1989, 1999) determine the activation of a feature set of the referent for each chosen word. The activation is computed as the sum of the associations of the individual features previously seen with each word. These competition-based models exhibit mutual exclusivity. All these models, both associative and competition-based, use toy datasets. Other models neither incorporate these phenomena nor exhibit any of them which makes them less biologically-plausible.

Social cues and cross-situational learning play a crucial role in learning word meanings (Gleitman, 1990; Baldwin, 1993; Tomasello and Akhtar, 1995; Pinker, 2013). To the best of our knowledge, no model that uses subsymbolic sensory representation of audio and visual data (e.g., Roy and Pentland, 2002; Yu and Ballard, 2004; Mangin and Oudeyer, 2013; Chen and Filliat, 2015; Räsänen and Rasilo, 2015) integrates social cues with cross-situational learning. Among symbolic audio-visual models (e.g., Yu and Ballard, 2007; Frank et al., 2008; Fontanari et al., 2009; Kachergis et al., 2012a; Kievit-Kylar et al., 2013), only Yu and Ballard (2007) has taken social pragmatic theory into account. The model in Lazaridou et al. (2016) considers visual attention and ignores prosodic information as it uses subsymbolic visual but symbolic audio representation. The symbolic data-based hybrid model in Kievit-Kylar et al. (2013) provides state-of-the-art F-score for object-referent learning; however, it does not consider any language-learning mechanism or social cues. Word-referent learning is still considered an open problem.

### 1.2. Contributions

The strong connection between reinforcement and the brain, particularly memory, is widely known (Schultz, 1998; Kirsch et al., 2003). It is proposed in recent theoretical models that integration of emerging language-learning mechanisms with phylogenetically older subcortical reward systems reinforce human motivation to learn a new language (Syal and Finlay, 2011; Ripollés et al., 2014). It is suggested from behavioral and imaging experiments that the anticipation of reward can have a beneficial effect on word-learning based on reward-induced anxiety (Callan and Schweighofer, 2008). In Longano and Greer (2015), behavioral data analysis suggests that acquisition of conditioned reinforcers for both visual and auditory stimuli provide the foundation for the naming capability. They proposed that visual and auditory stimuli may have reinforcing properties independently. But to learn naming, both stimuli must act at the same time to select the observing reactions of looking and listening. fMRI studies in Ripollés et al. (2014, 2016) found that adult participants exhibited robust fMRI activation in the core brain region [ventral striatum (VS)] for reward processing while learning new words. Similar activation is observed for VS recruitment when participants are engaged in an independent reward task. From these results it can be demonstrated that self-monitoring of correct performance triggers intrinsic reward-related signals. These reward signals help to store new information into long-term memory of the midbrain (Ripollés et al., 2014). From a computational viewpoint, reinforcement learning provides an alternative solution to supervised learning models. An agent can discover the ground truth through reward and punishment in an interactive setting.

The effort of an agent for obtaining a reward can be measured as a value using a value system (Schultz, 2000). Value system is one of the important components of developmental cognitive systems which promotes the “brain” to establish a link between behavioral responses and external event (Begum and Karray, 2009; Merrick, 2017). Value system is defined in Merrick (2017) as follows: *A value system permits a biological brain to increase the likelihood of neural responses to selected external phenomena*. A number of machine learning algorithms capture the necessity of this learning process. Computational value systems include reinforcement-based artificial neural networks (Merrick, 2017) where each environmental state-action pair is associated with a value. In Najnin and Banerjee (2016, 2017), a value system based on internal reinforcement is used for motor skill acquisition in speech production. Unfortunately, such systems have never been used for the speech perception task of word-learning.

In this paper, we extend the reinforcement learning framework for word-learning. Both table-based algorithms (Q-learning, SARSA, SARSA-λ) and neural network-based algorithms (Q-NN, NFQ, DQN) (Sutton and Barto, 1998; Riedmiller, 2005; Mnih et al., 2015) are experimented with. Cross-situational learning and social cues are taken into account. The advantage of using neural networks over table-based methods is that the former regulates reinforcement learning with superior generalization and convergence in real-world applications (Sutton and Barto, 1998; Shiraga et al., 2002). Social cues are extracted from joint attention and prosodic cues from the caregiver's speech. When only joint attention is used, the models are named as Attentional Q-learning, SARSA, SARSA-λ, Q-NN, NFQ, DQN. When both joint attention and prosodic cues are taken into account, they are named as Attentional-prosodic Q-learning, etc. The mother-infant interaction-based CHILDES dataset (MacWhinney, 2000) is used for experimentation. Evaluation of the proposed models follow similar procedures as in Frank et al. (2008), Kievit-Kylar et al. (2013), and Lazaridou et al. (2016).

Our experimental results show that reinforcement learning models are well-suited for word-learning. In particular, we show that:

- Word-object pairs can be learned using reinforcement.
- Attentional Q-NN, NFQ, DQN and their attentional-prosodic counterparts can select referent objects with high accuracy for given target words.
- Attentional-prosodic Q-NN, NFQ, and DQN outperform their attentional counterparts in terms of F-score, precision and recall.
- Attentional-prosodic DQN outperforms some of the prominent existing models in terms of F-score.

The rest of this paper is organized as follows. Section 2 covers how reinforcement learning can be extended for word-learning. The dataset and its complexity are described in Section 3. Section 4 details the experimental results. Finally, the paper ends with concluding remarks.

## 2. Learning word-object pairs through reinforcement

**Figure 2:**
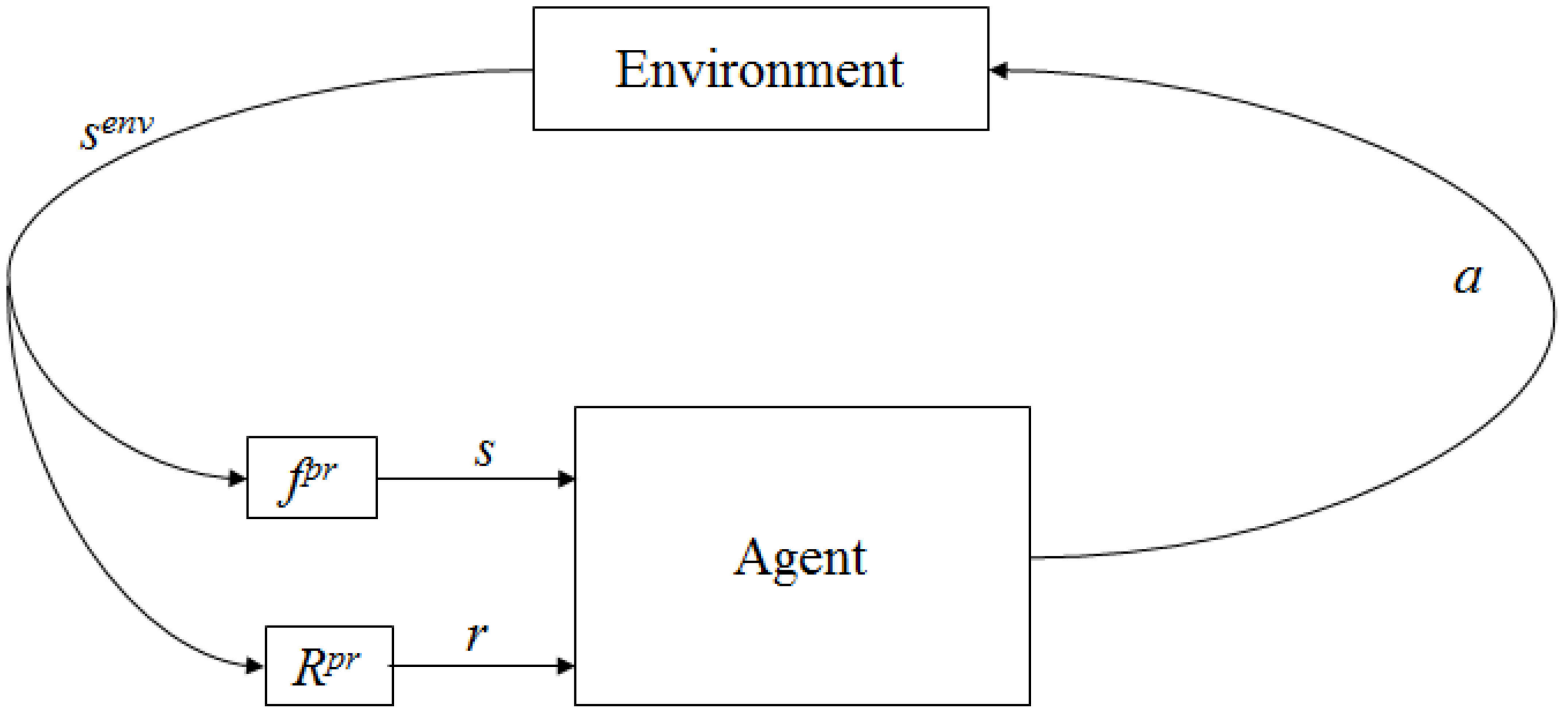
Agent-environment interaction in reinforcement learning. Here, *f*^*pr*^ indicate environment state (*s*^*env*^) is perceived by the agent as *s* through some process *f*^*pr*^ and *R*^*pr*^ represents the process of reward computation.

The proposed solution to the computational problem of learning word meanings rests on recent advances in reinforcement learning algorithms. An agent interacts with its environment via perception and action, as shown in Figure 2 (Kaelbling et al., 1996). At any instant, it receives a stimulus (*s*), some indication of the current state (*s*^*enυ*^) of the environment. It then chooses an action (*a*) to change the state of the environment. It receives a reward (*r*) based on the state transition. The agent is required to learn a policy (π) in order to choose a sequence of actions that is expected to maximize long-term sum of rewards.

Let *X* = {*x*_1_, *x*_2_, …, *x*_*N*_} be a word set that constitutes a vocabulary, and *O* = {*o*_1_, *o*_2_, …, *o*_*M*_} be a object/meaning set, where *N* is the number of acoustic words and *M* is the number of objects. In a caregiver-agent interactive scenario, each spoken utterance and the corresponding visual context formed one learning situation (Yu and Ballard, 2007). Extra-linguistic context includes objects present in the scene and social cues. The entire dataset contains multiple instances of such learning situations. Let *S* be the set of learning situations. In the *i*-th learning situation, the utterance *U*^*i*^ consists of *d* acoustic words, $\left\{u_{1}^{i},u_{2}^{i},\ldots ,u_{d}^{i}\right\}$, the meaning can be any of the *l* presented objects in the scene, $o^{i}=\left\{o_{\upsilon_{1}}^{i},o_{\upsilon_{2}}^{i},\ldots ,o_{\upsilon_{l}}^{i}\right\}$, υ_*j*_ ∈ {1, 2, …, *M*} ∀*j*. The attended object in the scenario is $o_{k}^{i}$, *k* ∈ {υ_1_, υ_2_, …, υ_*l*_}, υ_*j*_ ∈ {1, 2, …, *M*} where υ_*j*_ is the index of the object. So all word-object pairs are in a set *S* = {*U*^*i*^, *o*^*i*^}, and all word-attended-object pairs are in a set $SA=\left\{U^{i},o_{k}^{i}\right\}$, *i* ∈ {1, 2, …, *P*}, *P* is the number of learning situations.

For learning word meanings, the proposed agent is assumed to be embedded in an environment consisting of audio and visual stimuli. In any situation, the agent hears the caregiver's utterance (consisting of words) and sees the objects present in its immediate environment. In this paper, each state consists of spoken utterance and attended object in a situation. At current situation (*S*^*i*^), the agent perceives the current state of the environment (*s*^*i*^) and selects an action (*a*^*i*^) that chooses a word from the utterance for the attended object ($o_{k}^{i}$) in the environment. In standard reinforcement learning, the agent acts externally on the environment, observes the next state and receives an external reward based on the state transition. The caregiver's utterances in any situation is independent of the agent's external action. That is, the agent's external action is not changing the environment. Choosing a word is the internal action of the agent that generates an internal reward for the agent.

Computation of the internal reward is a non-trivial problem. The intuition behind this reward function is borrowed from language-specific constraints to restrict large hypothesis spaces; this facilitates cross-situational learning. These language-specific constraints help the infant to learn word-object mappings during early language development (Markman, 1992). According to the principle of mutual exclusivity (Markman and Wachtel, 1988), every object has only one label. Principle of contrast states that infants resort to fill-the-gap bias whereby they find a word for an object with no known word (Clark, 1987). Moreover, the novel name-nameless category principle states that novel words link to novel objects (Golinkoff et al., 1994). In Tilles and Fontanari (2013), an adaptive learning algorithm is proposed that contains two parameters: (1) to regulate the associative reinforcement between concurrent words and referents based on confidence values of past trials, and (2) to regulate the non-associative inference process that handles mutual exclusivity bias and information of past learning events. In the proposed computational agent, reward computation follows the novel name-nameless category principle. If an object is changed from one situation to the next, the agent will receive a reward if it chooses different words for the two objects. In the same vein, the agent will be rewarded if it chooses the same word for the same objects in two consecutive situations.

Based on the above principle, the proposed agent perceives the next situation (*S*^*i*+1^), selects action (*a*^*i*+1^) and receives reward (*r*_*i*+1_) based on state transition and action. The reward function is:

(1) $$\begin{array}{r} r_{i+1}=\left\{ \begin{array}{c} 1,\text{ if }\left(o_{k}^{i}=o_{k}^{i+1}\wedge a^{i}=a^{i+1}\right)\vee \left(o_{k}^{i}\neq o_{k}^{i+1}\wedge a^{i}\neq a^{i+1}\right)\;\\ -1,\;\text{otherwise}\; \end{array}\right. \end{array}$$

The agent's objective is to compute a policy, π, that maps from state (*s*^*i*^) to action (*a*^*i*^) by maximizing some long-term measure of reinforcement. The following example illustrates the interaction between the agent and its environment using two consecutive situations, *S*^1^ and *S*^2^.

Situation *S*^1^:

*Utterance*: “ahhah look we can read books david”

*Objects present in the immediate environment*: {<book>, <bird>, <rattle>, <face>}

*Attended object in the immediate environment*: <book>

*Environment*: The caregiver utters a sentence consisting of seven words. So the agent has seven possible actions to choose a word that corresponds to the attended object.

*Agent*: Takes action 6 (which is choosing the word “book”) as the meaning of the attended object.

Situation *S*^2^:

*Utterance*: “its a look and see book”

*Objects present in the immediate environment*: {<book>, <bird>, <rattle>, <face>}

*Attended object in the immediate environment*: <book>

*Environment*: The caregiver utters a sentence (state) consisting of six words. So the agent has six possible actions to choose from.

*Agent*: Let us consider two possible cases. Case 1: Takes action 6 (“book”) as the meaning of the attended object. Case 2: Takes action 2 (“a”) as the meaning of the attended object.

*Reward*: Case 1: Receives a reward of 1 unit. Case 2: Receives a reward of −1 unit.

The above formulation reduces the problem to a finite Markov decision process (MDP) where each situation is a distinct state. Hence, standard reinforcement learning methods for MDPs can be deployed by assuming *s*^*i*^ as the state representation at the *i*-th situation. The goal of the agent is to select actions in a way that maximizes future rewards. Future rewards are discounted by a factor of γ per situation. The future discounted reward at the *i*-th situation is defined as Sutton and Barto (1998):

(2) $$\begin{array}{r} R_{i}=\overset{P}{\underset{j=i}{\sum }}\gamma^{j-i}r_{j} \end{array}$$

For the task of learning word-object pairs, both table-based and neural network-based reinforcement learning methods will be investigated in this paper. Table-based methods include Q-learning, SARSA, and SARSA-λ. The neural network-based methods, namely Q-NN, NFQ, and DQN, will be extended for the word-learning task. Our formulation of these methods for the task is described in the following sections.

### 2.1. Q-learning

A number of reinforcement learning methods estimate the action-value function using the Bellman equation (Sutton and Barto, 1998). The optimal action-value function Q^*^(*s, a*) is defined as Kaelbling et al. (1996):

(3) $$\begin{array}{r} Q^{*}\left(s,a\right)=max_{\pi }𝔼\left[R_{i}|s_{i}=s,a_{i}=a,\pi \right] \end{array}$$

where Q^*^(*s, a*) is the maximum expected reward achievable by any strategy on perceiving situation *s* and then taking action *a*. π is a policy mapping states to actions.

The optimal action-value function obeys Bellman equation. If the optimal value Q^*^(*s*′, *a*′) of the sequence *s*′ at the next time-step is known for all possible actions *a*′, the optimal strategy is to select the action that maximizes the expected value of *r*+Q^*^(*s*′, *a*′) (Kaelbling et al., 1996). That is,

(4) $$\begin{array}{r} Q^{*}\left(s,a\right)=𝔼\left[r+\gamma max_{a^{\prime }}Q^{*}\left(s^{\prime },a^{\prime }\right)|s,a\right] \end{array}$$

Such action-value function can be estimated iteratively as Watkins and Dayan (1992):

(5) $$\begin{array}{r} Q_{i+1}\left(s,a\right)=𝔼\left[r+\gamma max_{a^{\prime }}Q_{i}\left(s^{\prime },a^{\prime }\right)|s,a\right] \end{array}$$

Convergence of such value iteration algorithms leads to the optimal action-value function, $Q_{i}\to Q^{*}$ as *i* → ∞ (Sutton and Barto, 1998). In classical Q-learning, the update rule is given by Sutton and Barto (1998), Kaelbling et al. (1996), and Watkins and Dayan (1992):

(6) $$\begin{array}{r} Q\left(s,a\right)\leftarrow Q\left(s,a\right)+\alpha \left(r+\gamma max_{a^{\prime }}Q\left(s^{\prime },a^{\prime }\right)-Q\left(s,a\right)\right) \end{array}$$

where α is a learning rate that decreases with iterations for convergence and γ is a discounting factor. It can be shown that, multiple updates of every state-action pair lead Q-learning to converge for finite state-action spaces (Sutton and Barto, 1998; Even-Dar and Mansour, 2003). Then, it results in optimal Q-function. Generally, the update is performed online in a sample-by-sample manner. That is, the value function is updated after every new transition. Q-learning is an off-policy algorithm (Even-Dar and Mansour, 2003). The current state consists of the utterance (*U*) and objects present in the agent's immediate environment in the current situation. Each utterance is a set of words *w* ∈ ℜ^*N*^. Since the number of situations is *P*, the Q-table is of size ℜ^*P*×*N*^. The *Q*-value for each word-object pair is computed as the running average of Q-values when an object is visited. In this way, a Q-matrix *QW* ∈ ℜ^*M*×*N*^ is constructed for word-object pairs. The Q-learning steps are shown in Algorithm 1.

**Algorithm 1:** Q-learning Algorithm for Word-Learning

1: Input: s is the current state, a is the current action, s′ is the next state, *r* is the immediate reward received
2: Initialize action-value function, *Q* table as randomly
3: Initialize Q-matrix for word-object pairs, *QW* as zeros
4: **for** *eachEpisode* = 1 *to Maxepisode* **do**
5: Observe current state, *s* = {*w, o*}
6: **for** *eachsituation* = 1 *to P* **do**
7: Choose action, *a* with probability ϵ using ϵ-greedy policy
8: Observe next state, *s*′, select a action, $a^{\prime }=max_{a^{\prime }}$ *Q*(*s*′, *a*′)
9: **if** (*o* = *o*′ and *a* = *a*′) or (*o* ≠ *o*′ and *a* ≠ *a*′) **then**
10: r = 100
11: **else**
12: r = −1
13: **end if**
14: **Update Qtable:**
15: $Q\left(s,a\right)\leftarrow Q\left(s,a\right)+\alpha \left(r+\gamma max_{a^{\prime }}Q\left(s^{\prime },a^{\prime }\right)-Q\left(s,a\right)\right)$
16: **Construct Q-matrix for word-object pairs**, *QW*:
17: **for** *eachobject, j* = 1 *to M* **do**
18: **if** *label*(*o*) = *j* **then**
19: *count* ← *count* + 1
20: $QW_{j}\leftarrow QW_{j}+\frac{Q\left(s,a\right)-QW_{j}}{count}$
21: **end if**
22: **end for**
23: *s* ← *s*′
24: **end for**
25: **end for**

### 2.2. SARSA

State-Action-Reward-State-Action (SARSA) is an on-policy algorithm for temporal difference learning and is more realistic than Q-learning. According to Russell and Norvig (2002), *if the overall policy is even partly controlled by other agents, it is better to learn a Q-function for what will actually happen rather than what the agent would like to happen*. The key difference from Q-learning is that Q-values are updated with new action and reward instead of using the maximum reward of the next state (Sutton and Barto, 1998). The Q-matrix for word-object pairs, *QW*, is constructed in the same way as in Q-learning. The SARSA steps are shown in Algorithm 2.

**Algorithm 2:** SARSA Algorithm for Word-Learning

1: Input: States, *S*
2: Initialize action-value function, *Q* table randomly
3: Initialize Q-matrix for word-object pairs, *QW* as zeros
4: **for** *eachEpisode* = 1 *to Maxepisode* **do**
5: Observe current state, *s* = {*w, o*}
6: **for** *eachsituation* = 1 *to P* **do**
7: Choose action, *a* with probability ϵ using ϵ-greedy policy
8: Observe next state, *s*′, select action, *a*′ with probability ϵ using ϵ-greedy policy
9: **if** (*o* = *o*′ and *a* = *a*′) or (*o* ≠ *o*′ and *a* ≠ *a*′) **then**
10: r = 100
11: **else**
12: r = −1
13: **end if**
14: **Update Qtable:**
15: *Q*(*s, a*) ← *Q*(*s, a*) + α(*r* + γ*Q*(*s*′, *a*′) − *Q*(*s, a*))
16: **Construct Q-matrix for word-object pairs**, *QW*:
17: **for** *eachobject, j* = 1 *to M* **do**
18: **if** *label*(*o*) = *j* **then**
19: *count* ← *count* + 1
20: $QW_{j}\leftarrow QW_{j}+\frac{Q\left(s,a\right)-QW_{j}}{count}$
21: **end if**
22: **end for**
23: *s* ← *s*′, *a* ← *a*′
24: **end for**
25: **end for**

### 2.3. SARSA-λ

The SARSA-λ algorithm was experimented with to observe the effect of memory in word-learning. Adding eligibility traces to the SARSA algorithm forms SARSA-λ algorithm (Loch and Singh, 1998). In this algorithm, n-steps backup are carried out instead of one step backup in SARSA or Q-learning. The value of λ determines the value of *n*. An eligibility trace acts as an additional memory variable for every state-action pair (Sutton and Barto, 1998). Q-matrix for word-object pairs, *QW*, is constructed in the same way as in Q-learning. The SARSA-λ steps are shown in Algorithm 3.

**Algorithm 3:** SARSA-λ Algorithm for Word-Learning

1: Input: States, *S*
2: Initialize action-value function, *Q*(*s, a*) randomly and eligibility trace, *e*(*s, a*) = 0 for all *s*, *a*
3: Initialize Q-matrix for word-object pairs, *QW* as zeros
4: **for** *eachEpisode* = 1 *to Maxepisode* **do**
5: Observe current state, *s* = {*w, o*}
6: for *eachsituation* = 1 *to P* **do**
7: Choose action, *a* = *max*_*a*_*Q*(*s, a*)
8: Observe next state, *s*′, select action, *a*′ with probability ϵ using ϵ-greedy policy
9: **if** (*o* = *o*′ and *a* = *a*′) or (*o* ≠ *o*′ and *o* ≠ *o*′) **then**
10: r = 100
11: else
12: r = −1
13: **end if**
14: δ = *r* + γ*Q*(*s*′, *a*′) − *Q*(*s, a*)
15: *e*(*s, a*) ← 1
16: **Update Q-table and eligibility traces:**
17: *Q*(*s, a*) ← *Q*(*s, a*) + αδ*e*(*s, a*)
18: *e*(*s, a*) ← γλ*e*(*s, a*)
19: **Construct Q-matrix for word-object pairs**, *QW*:
20: **for** *eachobject, j* = 1 *to M* **do**
21: **if** *label*(*o*) = *j* **then**
22: *count* ← *count* + 1
23: $QW_{j}\leftarrow QW_{j}+\frac{Q\left(s,\;a\right)-QW_{j}}{count}$
24: **end if**
25: **end for**
26: *s* ← *s*′, *a* ← *a*′
27: **end for**
28: **end for**

### 2.4. Q-learning using neural networks

In this paper, the state of the environment in the word-learning case is defined as a situation consisting of utterances and objects presented at that time. In the real world, children below three years of age hear at least 240 utterances per hour (Grabe and Stoller, 2013)-p70. In a year, they encounter approximately 21 million utterances. So there will be 21 million rows in our imaginary Q-table in the table-based method which is computationally inefficient for the Q-table to converge. Ideally, a good prediction for *Q*-values is required for unseen states. Neural networks are good at learning useful features for highly-structured data. Therefore, Q-function could be represented by a neural network where state is the input and *Q*-value for each possible action are the outputs. The advantage of this approach is that, performing a *Q*-value update or choosing the action with highest *Q*-value requires only one forward pass through the network. Table-based Q-learning is not practical because of separate action-value function estimation process for each situation without any generalization capability.

In neural network-based method, an error function is introduced that measures the difference between the current *Q*-value and the new value that should be assigned. For example, a squared-error function may be used: $error={\left(Q\left(s,a\right)-\left(r+\gamma max_{a^{\prime }}Q\left(s^{\prime },a^{\prime }\right)\right)\right)}^{2}$ (Riedmiller, 1999). The Q-learning rule in Equation (6) can be directly implemented as a neural network minimizing this squared-error. A neural network function approximator with weights θ will be referred to as a Q-network (Sutton and Barto, 1998). Gradient descent techniques, such as the backpropagation learning rule, can be applied to update the weights of a neural network by minimizing the error (Baird and Moore, 1999). The Q-matrix, *QW*, for word-object pairs is computed using running average in the same way as in Q-learning. The steps are shown in Algorithm 4.

**Algorithm 4:** Q-learning with Neural Network for Word-Learning

1: Initialize Q-matrix for word-object pairs, *QW* as zeros
2: Initialize action-value function, *Q* with random weight, θ
3: Learning rate, α = 0.001, discount rate, γ = 0.99
4: Initialize ϵ = 0.99 for ϵ − *greedy* action selection.
5: **for** *eachEpisode* = 1 *to Maxepisode* **do**
6: Observe current state, *s* = {*w, o*}
7: **for** *eachsituation* = 1 *to P* **do**
8: Calculate *Q* using perceptron network with two hidden layers, *Q* = *net*(*s*, θ)
9: Choose action, *a* using ϵ-*greedy* policy.
10: Observe *s*′, choose *a*′ based on maximum *Q*-value.
11: Calculate reward *r* as described in Algorithm.1.
12: Set $Q_{tr}=r_{j}+\gamma Q\left(s^{\prime },a^{\prime },\theta \right)$
13: Perform gradient descent step on ${\left(Q_{tr}-Q\left(s,a,\theta \right)\right)}^{2}$ with respect to the network parameter θ
14: Calculate *Q* − *value* for state *s* with *Q*_*up*_ = *net*(*s*, θ)
15: **Construct Q-matrix for word-object pairs**, *QW*:
16: **for** *eachobject, j* = 1 *to M* **do**
17: **if** *label*(*o*) = *j* **then**
18: *count* ← *count* + 1
19: $QW_{j}\leftarrow QW_{j}+\frac{Q_{up}-QW_{j}}{count}$
20: **end if**
21: **end for**
22: Set *s* ← *s*′;
23: **end for**
24: Decrease ϵ linearly.
25: **end for**

### 2.5. Neural-fitted Q-network

Q-learning for neural network is an online algorithm where Q-network parameters (θ) are typically updated after each new situation. The problem with the online update rule is that, typically a large number of iterations is required to compute an optimal or near-optimal policy (Riedmiller, 1999). If weights are tuned for a state-action pair, undesirable changes might occur at other state-action pair which, in our experience, is the primary reason behind slow learning (Riedmiller, 1999). NFQ is an example of the Fitted-Q Iteration family of algorithms (Ernst et al., 2005) where multilayer perceptron network is used for regression. The steps are shown in Algorithm 5. It consists of two major steps: generating the training set *D* and training a multilayer perceptron using these patterns.

**Algorithm 5:** NFQ-Algorithm for Word-Learning

1: Initialize Q-matrix for word-object pairs, *QW* as zeros
2: Initialize a set of transition samples, *D* to capacity *J*
3: Initialize action-value function, *Q* with random weight, θ
4: Learning rate, α = 0.001, discount rate, γ = 0.99
5: Initialize ϵ = 0.99 for ϵ-*greedy* action selection.
6: **for** *eachEpisode* = 1 *to Maxepisode* **do**
7: Observe current state, *s* = {*w, o*}
8: for *eachsituation* = 1 *to P* **do**
9: Calculate *Q* using perceptron network with two hidden layers, *Q* = *net*(*s*, θ)
10: Choose action, *a* using ϵ − *greedy* policy.
11: Observe *s*′, choose *a*′ based on maximum *Q*-value.
12: Calculate reward *r* as described in Algorithm.1.
13: Store transition (*s*, *a*, *s*′, *a*′, *r*) as (*s*_*j*_, *a*_*j*_, *s*_*j*+1_,*a*_*j*+1_, *r*_*j*_) in *D* where *j* is 1 to *J* − 1.
14: Set the target Q-value for current state as: *Q*_*tr*_ = *r*_*j*_ + γ*Q*(*s*_*j*+1_, *a*_*j*+1_, θ)
15: Perform gradient descent step on ${\left(Q_{tr}-Q\left(s_{j},a_{j},\theta \right)\right)}^{2}$ with respect to the network parameter θ
16: Calculate *Q* − *value* for state *s* with *Q*_*up*_ = *net*(*s*, θ)
17: **Construct Q-matrix for word-object pairs**, *QW*:
18: **for** *eachobject, jj* = 1 *to M* **do**
19: **if** *label*(*o*) = *jj* **then**
20: *count* ← *count* + 1
21: $QW_{jj}\leftarrow QW_{jj}+\frac{Q_{up}-QW_{jj}}{count}$
22: **end if**
23: **end for**
24: Set *s* ← *s*′;
25: **end for**
26: Decrease ϵ linearly.
27: **end for**

### 2.6. Deep-Q learning

In deep-Q learning, an agent's experience at each situation is stored in a replay memory, termed as *experience replay* (Mnih et al., 2015). This is an important trick for training neural networks where random mini-batches are drawn from replay memory instead of most recent experience for updating the Q-network. This random choice breaks similarity between subsequent situations which reduces the variance of the updates (Mnih et al., 2015). Moreover, during training, multiple weight updates in each situation lead to higher data efficiency in DQN over Q-NN (Van Hasselt et al., 2016). The agent selects and executes an action according to an ϵ-greedy policy after performing experience replay (Mnih et al., 2015). The steps are shown in Algorithm 6.

**Algorithm 6:** Double deep Q learning for optimal control

1: Initialize experience replay memory *D* to capacity *J*
2: Initialize action-value function, *Q* with random weight, θ
3: Initialize target action value function, *Q*_*tr*_ with random weights, θ^*p*^ = θ.
4: Learning rate, α = 0.001, discount rate, γ = 0.99
5: Initialize ϵ = 0.99 for ϵ-*greedy* action selection.
6: **for** *eachEpisode* = 1 *to Maxepisode* **do**
7: Observe current state, *s* = {*w, o*}
8: **for** *eachsituation* = 1 *to P* **do**
9: Calculate *Q* using perceptron network with two hidden layers, *Q* = *net*(*s, a*, θ)
10: With probability ϵ select random acrion *a*
11: otherwise select *a* = *argmax*_*a*_*Q*
12: Observe *s*′, choose *a*′ based on maximum $\hat{Q}$-value, $\hat{Q}=net\left(s^{\prime },a^{\prime },\theta^{p}\right)$.
13: Calculate reward *r* as described in Algorithm.1.
14: Store transition (*s*, *a*, *s*′, *a*′, *r*) in *D*.
15: Sample minibatch of transitions (*s*_*j*_, *a*_*j*_, *s*_*j*+1_,*a*_*j*+1_, *r*_*j*_,) from *D*
16: Set $Q_{tr}=r_{j}+\gamma \underset{a^{\prime }}{\arg\max}\left(s_{j+1},a^{\prime },\theta^{p}\right)$
17: Perform gradient descent step on ${\left(Q_{tr}-Q\left(s_{j},a_{j},\theta \right)\right)}^{2}$ with respect to the network parameter θ
18: After *C* step reset *Q*_*tr*_ network with *Q* by setting θ^*p*^ = θ
19: Calculate *Q* − *value* for state *s* and chosen action, *a* with *Q*_*up*_ = *net*(*s, a*, θ)
20: **Construct Q-matrix for word-object pairs**, *QW*:
21: **for** *eachobject, jj* = 1 *to M* **do**
22: **if** *label*(*o*) = *jj* **then**
23: *count* ← *count* + 1
24: $QW_{jj}\leftarrow QW_{jj}+\frac{Q_{up}-QW_{jj}}{count}$
25: **end if**
26: **end for**
27: Set *s* ← *s*′;
28: **end for**
29: Decrease ϵ linearly.
30: **end for**

## 3. Dataset

Two transcribed video clips (me03 and di06) of mother-infant interaction from the CHILDES dataset (MacWhinney, 2000) is used in this study, as in Yu and Ballard (2007), Frank et al. (2008), Kievit-Kylar et al. (2013), and Lazaridou et al. (2016). Each recording is approximately of 10 min duration. These clips represent an environment where a mother introduces a pre-verbal infant to a set of toys. Each utterance is annotated manually in the transcripts. Transcriptions consist of a list of object labels (e.g., ring, hat, cow) corresponding to objects present in the immediate environment of the infant while the utterance took place. A bidirectional relationship between mother and infant involves seeing, hearing, touching, and pointing. Social cues encoded in multimodal interaction help young language learners to learn word-object relations (Yu and Ballard, 2007). In Frank et al. (2008), social cues from infant's eyes, hands, mouth, touch, and mother's hands, eyes, touch are considered. In Yu and Ballard (2007), two social cues, visual joint-attention cues and prosodic cues in infant-directed speech, are taken into account.

Several aspects make the CHILDES dataset challenging for learning word-object relations. In this type of bidirectional natural multimodal interaction, the vocabulary is large and the number of object labels are very less in comparison to the most frequent words. In these videos, only 2.4 and 2.7% co-occurring word-object pairs are relevant while rest are irrelevant. Moreover, this dataset contains a large amount of referential uncertainty where for each utterance up to seven objects are presented in the scene. Mother uttered the name an attended object explicitly only in 23% of utterances. Putting these facts together, there are many frequent but irrelevant pairs consisting of function words and a small set of referent objects. Thus, learning word-to-referent object mappings by computing co-occurrence frequencies leads to incorrect associations. Considering the smoothness and efficiency in word-learning, it is highly probably that infants use a more smart and effective strategy to learn relevant word-referent object pairs.

## 4. Experiments

### 4.1. Experimental setup

#### 4.1.1. Data representation

Each of the models discussed in section 2 is trained using a subset of CHILDES dataset (MacWhinney, 2000). The results are compared to the models in Frank et al. (2008), Kievit-Kylar et al. (2013), and Lazaridou et al. (2016). The dataset has two streams: audio and video. The video stream is represented as symbolic channels and consists of a list of object labels. While an utterance is occurring, all mid-size objects in view are labeled and included in the list. The list grows from empty to a set of object labels. For audio stream, two cases are considered:

1. The audio stream is represented as a symbolic channel containing the words that occurred in a sentence, as in Yu and Ballard (2007), Frank et al. (2008), and Chen and Filliat (2015). Using the transcriptions, the word-occurrence is coded as a binary vector of the size of the vocabulary of all known words. The vocabulary is created incrementally, starting from an empty set and including each new word encountered in sentences at the end.
2. The caregiver's speech is segmented into utterances based on speech silence. Each utterance is aligned with transcriptions using Sphinx speech recognition system (Lee et al., 1990). The time-stamps of the beginning and end of each spoken word are extracted. The prosodic features are computed for each word, as in Yu and Ballard (2007). The prosodic feature for each word in an utterance is computed as the difference between 75−*percentile pitch* of the word and the utterance. 75−*percentile pitch* of an utterance means the 75th percentile pitch of the utterance. So, this allows each word to be presented as a scalar prosodic feature value.

In this paper, two sets of experiments are done using social cues: (1) joint attention-based reinforcement learning where joint attention on the objects are considered as social cues and the audio stream is symbolically represented; (2) joint attention and prosodic cue-based reinforcement learning where both jointly attentive object and prosody in the caregiver's speech are considered as social cues. In the latter case, audio stream is represented as prosodic feature. For both experiments, video stream is represented as a list of object labels. The dataset consists of 624 utterances with 2,533 words in total. The vocabulary size is 419. The number of objects and referent objects are 22 and 17 respectively. The dataset includes a gold-standard lexicon consisting of 37 words paired with 17 objects (Frank et al., 2008; Kievit-Kylar et al., 2013; Lazaridou et al., 2016). The models have to discover word-referent object pairs.

#### 4.1.2. Joint attention

To find the object jointly attended by both caregiver and infant, we follow the same methodology as in Yu and Ballard (2007) based on body movement cues that indicate the speaker's visual attention. In Baldwin et al. (1996), experiments are performed on children of age 18–20 months under two cases: (Case 1) a word is uttered by a caregiver who is within the infant's view and concurrently directed attention toward the target toy, and (Case 2) a word is uttered by a caregiver who is outside of the infant's view. Infants can establish a stable link between the novel word and target object only in case 1. Same observation is found in Deák and Triesch (2006) which indicates that joint visual attention is an important factor in development and learning. Based on this observation, jointly-attended objects are manually identified from the video. In Yu and Ballard (2007), two categories of extra-linguistic contextual information for each learning situation are described. One category consists of objects jointly attended by the child and the caregiver. The second represents all the other objects in the visual field. A few examples of jointly-attended objects are as follows:

Example 1:
*Utterance: The kitty-cat go meow meow*.
Visual context: baby, big-bird, rattle, book
Attended object: kitty-cat

Example 2:
*Utterance: Yeah, I see those hands*.
Visual context: hand, big-bird
Attended object: hand

#### 4.1.3. Prosodic cues

In Snedeker and Trueswell (2003), it is shown that speakers produce and listeners use prosodic cues to distinguish between alternative meanings of a word in a referential interaction. It suggests that not only linguistic information (what is said, etc.) but also non-linguistic aspects of speech contain decisive information. Here, the linguistic information refers to what the speaker said and the non-linguistic information refers to how it is said. One role of prosodic cues in word-learning is to help young learners identify key words from the speech stream. It is shown in Yu and Ballard (2007) that prosodically-salient words in infant-directed maternal speech contain the most important linguistic information that the caregiver intends to convey. In Yu and Ballard (2007), it is shown through data analysis on CHILDES dataset that the word corresponding to the referrent/object has higher pitch than any other word in the infant-directed utterance. Moreover, pitch enhancement of the target word related to object helps to learn word-object pairs (Filippi et al., 2014). This motivates us to extract the values of 75−*percentile pitch* over both an utterance and the words within the utterance to obtain the prosodically-highlighted words in each spoken utterance. For the word *w*_*i*_ in the spoken utterance *u*_*j*_, we formed the feature: $p_{75}^{w_{i}}-p_{75}^{u_{j}}$. When using symbolic representation of auditory stream, the word-occurrence is coded as a binary vector of the size of the vocabulary of all known words. When considering prosody, instead of binary representation, word occurrence is coded by the above feature of the words in the utterance at that situation.

#### 4.1.4. Evaluation metrics

The performance of proposed models for the task of word-referent object pair learning are evaluated using the following criteria:

- Word-referent Matrix: If all 37 words are assigned an object, the proportion of pairings matching with the golden standard can be calculated from the confusion matrix representing the Word-referent Matrix (Kievit-Kylar et al., 2013). According to the gold standard, each word is associated with exactly one object except for “bird” which can refer to <bird>or <duck>. Based on this hypothesis, a winner-take-all filter has been applied for each gold-standard word to compute the confusion matrix in Kievit-Kylar et al. (2013). In the current paper, confusion matrix is determined by extracting Q-values of each gold-standard word for all the objects from Q-matrix, *QW*. To have the same scaling for all the objects, *QW* is first normalized and then normalized Q-matrix is used to compute confusion matrix. For each object *O*_*i*_, normalized Q-matrix (*QW*_*norm*_) and confusion matrix are computed as follows:
  (7) $$\begin{array}{r} QW_{norm}\left(O_{i}\right)=QW\left(O_{i}\right)/max\left(QW\left(O_{i}\right)\right) \end{array}$$
  (8) $$\begin{array}{r} confusion\;matrix=QW_{norm}\left(w_{gold},O_{i}\right) \end{array}$$
  where, *w*_*gold*_ is the set of gold-standard words and *i* is the index of the object. So, for a given *j*-th target word/gold-standard word (*w*_*gol*_*d*__*j*__), the referent object (*O*_*j*_) can be found as:
  (9) $$\begin{array}{r} O_{j}=argmax_{i}QW_{norm}\left(w_{{gold}_{j}},O_{i}\right) \end{array}$$
- Quality of learned word-referent lexicon (Yu and Ballard, 2007; Frank et al., 2008; Lazaridou et al., 2016): Three measures are used to evaluate the quality of lexicon learned using the proposed models. They are: (1) Precision (the percentage of words spotted by the model which are actually correct), (2) Recall (the percentage of correct words that the model learned among all the relevant words that are expected to be learnt), and (3) F-score (the harmonic mean of precision and recall).

Performance of the proposed models is compared to that of the existing models in Frank et al. (2008), Kievit-Kylar et al. (2013), and Lazaridou et al. (2016) and the baseline association model based on co-occurrence called COOC. Bayesian CSL is the original Bayesian cross-situational model (Frank et al., 2008), also including social cues. BEAGLE+PMI is the best semantic-space model across a range of distributional models and word-object matching methods which produces state-of-the-art F-score in learning word-object pairs (Kievit-Kylar et al., 2013).

### 4.2. Simulation results

#### 4.2.1. Training parameters

We investigated the word-learning performance in a virtual agent using models based on six reinforcement learning algorithms: Q-learning, SARSA, SARSA-λ, NFQ, and DQN. For all the cases, the learning rate (α), discount rate (γ) and ϵ are chosen empirically as 0.001, 0.99, and 0.99, respectively. For Q-learning, SARSA and SARSA-λ, Q-tables are initialized randomly. λ is chosen as 0.9 empirically for SARSA-λ.

For each neural network model for word-learning, the Q-network has four-layers with the first layer being the input layer, second and third layers are hidden, while the fourth is the output layer representing the *Q*-value of each word. So input and output layers are of dimension 419 each. The activation function in the two hidden layers is sigmoid. In the output layer, activation function is chosen as softmax which leads the agent to have higher *Q*-value for one word per utterance.

The neural network weights are initialized randomly in [10^−2^, 10^2^]. In this paper we have used online gradient decent algorithm for learning QNN and stochastic gradient descent for learning NFQ and DQN. For all three neural network-based algorithms, the behavior policy during training was ϵ-greedy with ϵ annealed linearly from 0.99 to 0.0001 and fixed at 0.0001 thereafter. The capacity *J* of storage *D* in NFQ network is set empirically to 50 for the result reported in this paper. For DQN, the capacity *J* of experience replay *D* is set to 50 of most recent experienced situations, mini-batch size is 10 and *C* = 4 (the target network is updated every four steps) in Algorithm 6.

#### 4.2.2. Word-learning using joint attention-based reinforcement learning

**Figure 3:**
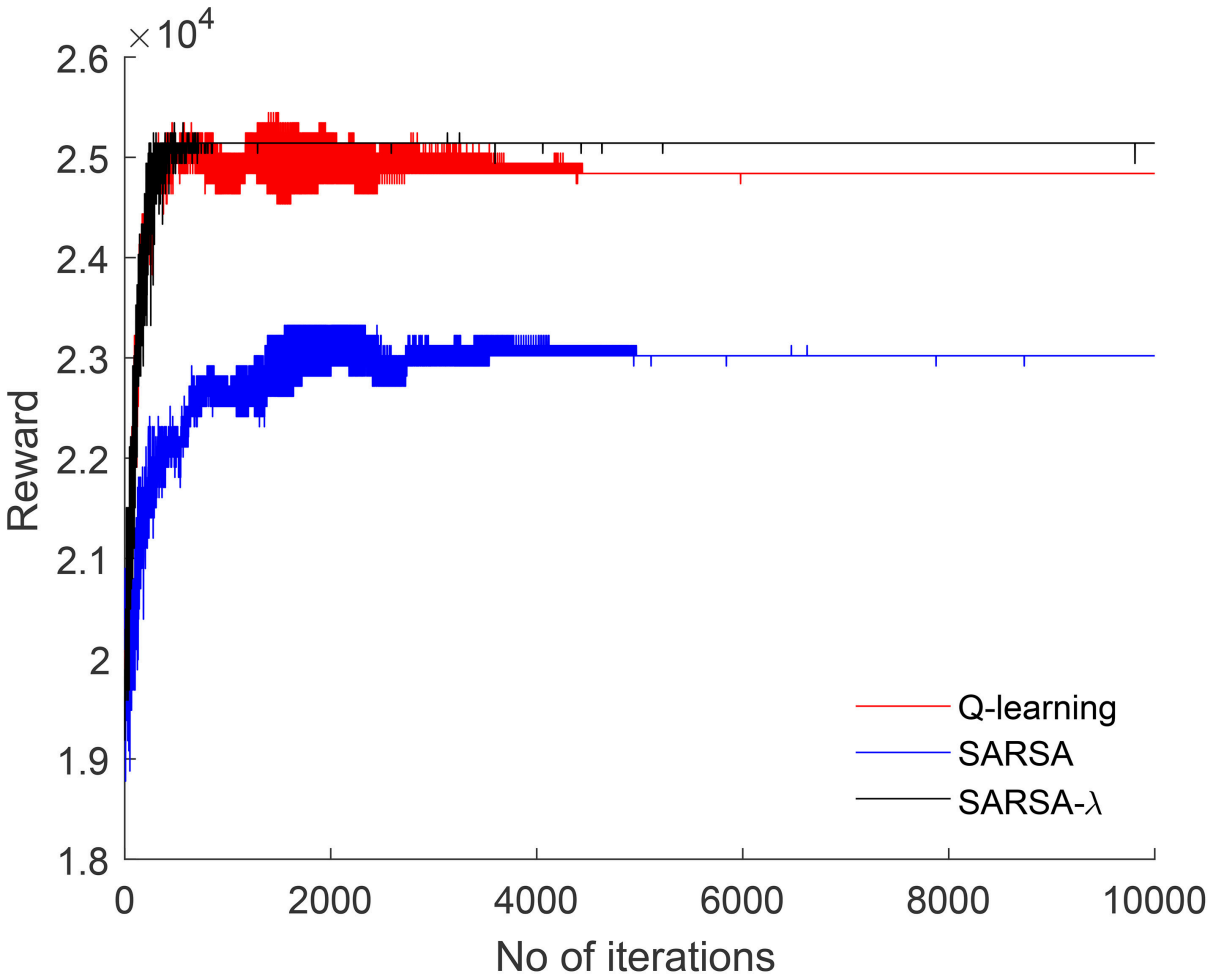
For each iteration, reward is computed and plotted for Q-learning, SARSA, and SARSA-λ, respectively.

**Figure 4:**
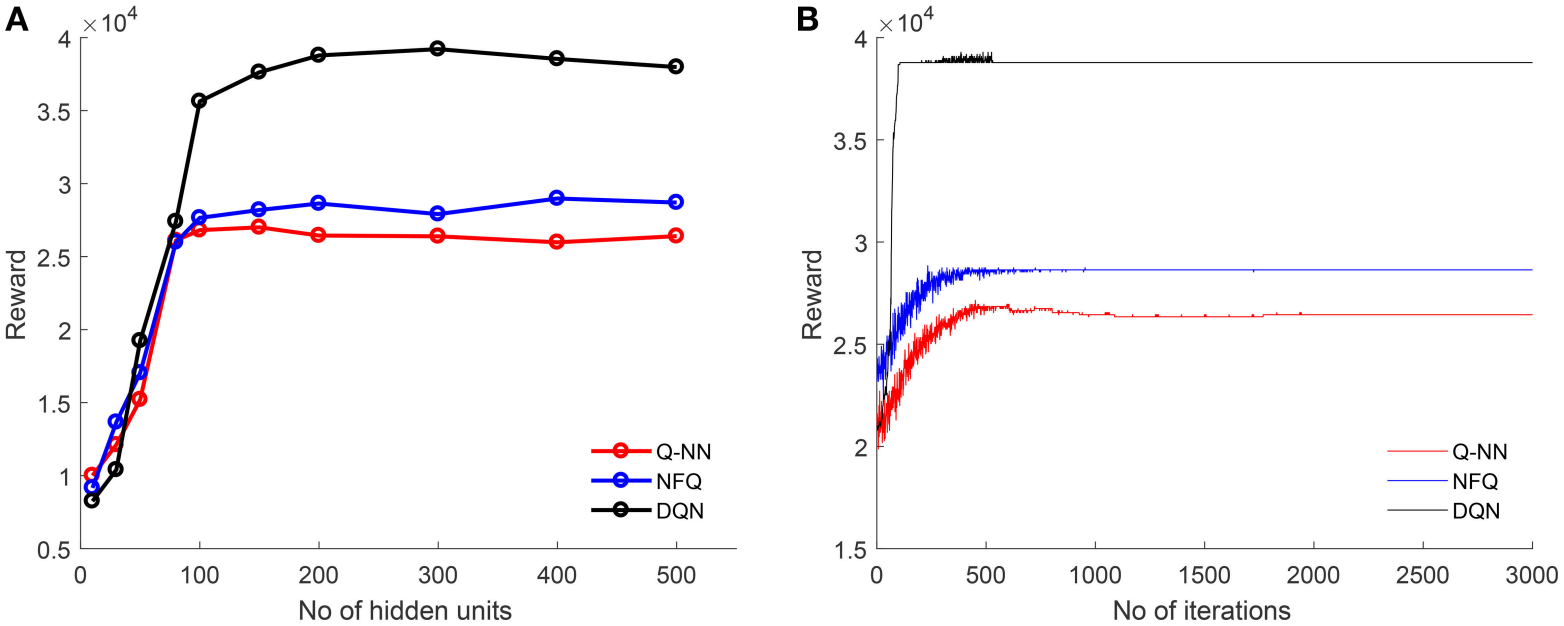
For Q-NN, NFQ, and DQN: **(A)** obtained maximum rewards for different number of hidden units are plotted to find the optimal structure of Q-network; **(B)** for each iteration, with optimal number of hidden unit (200 units) reward is computed and plotted.

The input to each model is the auditory stream represented as a binary vector. The reward for each situation is computed based on jointly attended object transition. Each table-based model (Q-learning, SARSA, SARSA-λ) is run for 10,000 iterations. Rewards computed in each iteration are shown in Figure 3. Q-learning and SARSA converge after 5,000 iterations whereas SARSA-λ converges sooner after 2,000 iterations. Total reward is higher in SARSA-λ than Q-learning or SARSA. In order to estimate the optimal structure of each neural network-based model, the number of hidden units is varied from 10 to 500, and rewards are computed for each case. Figure 4A shows the reward with respect to different number of hidden units for QNN, NFQ, and DQN. For any network, reward did not increase beyond 200 hidden units. So the neural network consists of 200 units in each hidden layer for QNN, NFQ, and DQN. Reward in DQN is greater than NFQ and Q-NN at convergence with the optimal neural network structure (ref. Figure 4B).

**Figure 5:**
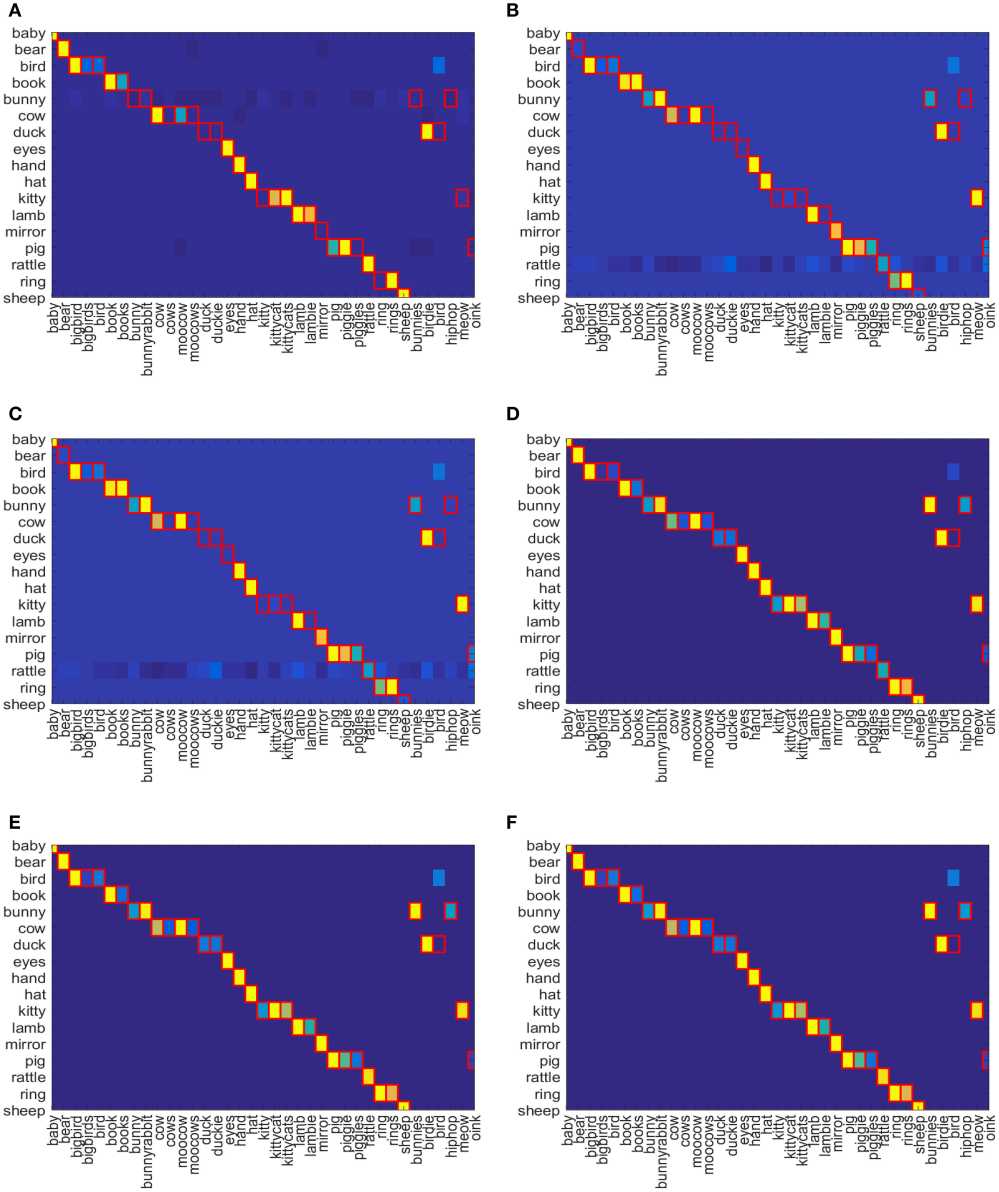
Confusion Matrix for **(A)** Attentional Q-learning, **(B)** Attentional SARSA, **(C)** Attentional SARSA-λ, **(D)** Attentional Q-NN, **(E)** Attentional NFQ, and **(F)** Attentional DQN respectively.

The result of word-referent pairs learning can be visualized using a color confusion matrix. The confusion matrix shows the similarity between word-object pairings as gradients from yellow to blue color filling each grid cell where yellow stands for higher *Q*-value and blue stands for lower. The cells outlined in red represent the correct word-object pairings in gold standard lexicon. The confusion matrix is computed using Equation (8) which consists of *Q*-values of all objects for each gold standard word for Attentional Q-learing, SARSA, SARSA-λ, Q-NN, NFQ, DQN and depicted in Figures 5A–F. The object that has the highest association is assigned the higher *Q*-value (colored yellow). The color gradually moves from yellow to blue as *Q*-value decreases. The *Q*-values returned by the reinforcement learning algorithms cannot be directly interpreted as probabilities for pairing selections. Hence, only relative similarity measures are used here. For each reinforcement model and each gold-standard word, Equation (9) is applied on confusion matrix to get the associated object related with the gold-standard word. The results are shown in Table 1 where the gold-standard words that are associated with wrong objects are mentioned. Table 1 shows that table-based methods, Q-learning, SARSA, and SARSA-λ, identified 14, 12, and 7 word-object pairs incorrectly given the target words. In comparison, all the neural network-based methods correctly identified 36 word-object pairs out of 37. None of the methods was able to relate two words to one object. All of them chose “bird” to refer to <bird>only when it can also be associated with the object <duck>.

**Table 1:** Gold-standard words incorrectly associated with gold-objects using Q-learning, SARSA, SARSA-λ, Q-NN, NFQ, and DQN.

**Q-learning**	**SARSA**	**SARSA-λ**	**Q-NN**	**NFQ**	**DQN**
“bunny”	“bigbirds”	“moocows”	“bird”	“bird”	“bird”
“cows”	“bunnyrabbit”	“duck”			
“moocows”	“cows”	“duckie”			
“duck”	“moocows”	“kittycats”			
“duckie”	“duckie”	“lambie”			
“kitty”	“kitty”	“bird”			
“mirror”	“kittycats”	“hiphop”			
“piggies”	“lamb”				
“ring”	“lambie”				
“bunnies”	“bunnies”				
“bird”	“bird”				
“hiphop”	“oink”				
“meow”					
“oink”					

**Figure 6:**
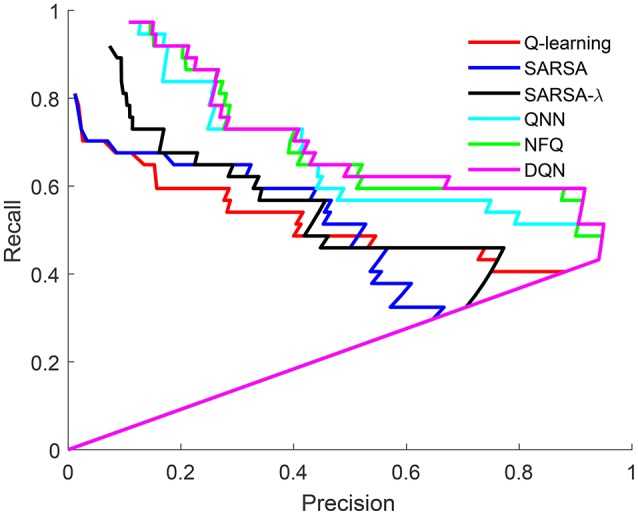
ROC for Q-learning, SARSA, SARSA-λ, Q-NN, NFQ, and DQN, respectively.

It is important to note which word/object pairs are more or less likely to be discovered by the model. A lexicon is created from the *Q*-value matrix based on a threshold, as in Frank et al. (2008). Figure 6 shows the precision and recall for lexicons across the full range of threshold values for Q-learning, SARSA, SARSA-λ, Q-NN, NFQ, and DQN for the task of word-learning. Unlike standard receiver operating characteristic (ROC) curves for classification tasks, the precision and recall of a lexicon depends on the entire lexicon. The irregularities in the curves in Figure 6 reflect the small size of the lexicons. For each of the learned models, the best F-score, precision and recall are tabulated in Table 2. For word-learning, neural network-based models (Attentional Q-NN, NFQ, DQN) have comparatively higher F-score and precision than table-based models (Attentional Q-learning, SARSA, SARSA-λ). Though the precision is highest for Attentional Q-NN, highest F-score is achieved by Attentional DQN due to higher recall than Attentional Q-NN.

**Table 2:** F-score, precision, and recall values using the learned lexicon from Attentional reinforcement learning models.

	**F-score**	**Precision**	**Recall**
Attentional Q-learning	0.5667	0.7391	0.4595
Attentional SARSA	0.5205	0.5278	0.5135
Attentional SARSA-λ	0.5763	0.7727	0.4595
Attentional Q-NN	0.6667	**0.95**	0.5135
Attentional NFQ	0.7097	0.88	**0.5946**
Attentional DQN	**0.7213**	0.9167	**0.5946**

*The bold values in each column indicate highest score over the algorithms*.

#### 4.2.3. Word-learning using joint attention-prosody-based reinforcement learning

As neural network-based reinforcement learning models outperform table-based models, simulations were run on the former for word-referent learning considering audio and visual social cues: prosody and joint attention. The input to each model is the audio stream represented by prosodic feature vector instead of binary vector. The reward for each situation is computed based on jointly attended object transition as described in section 2. The same optimal structure of the Q-network from the last section is retained.

**Figure 7:**
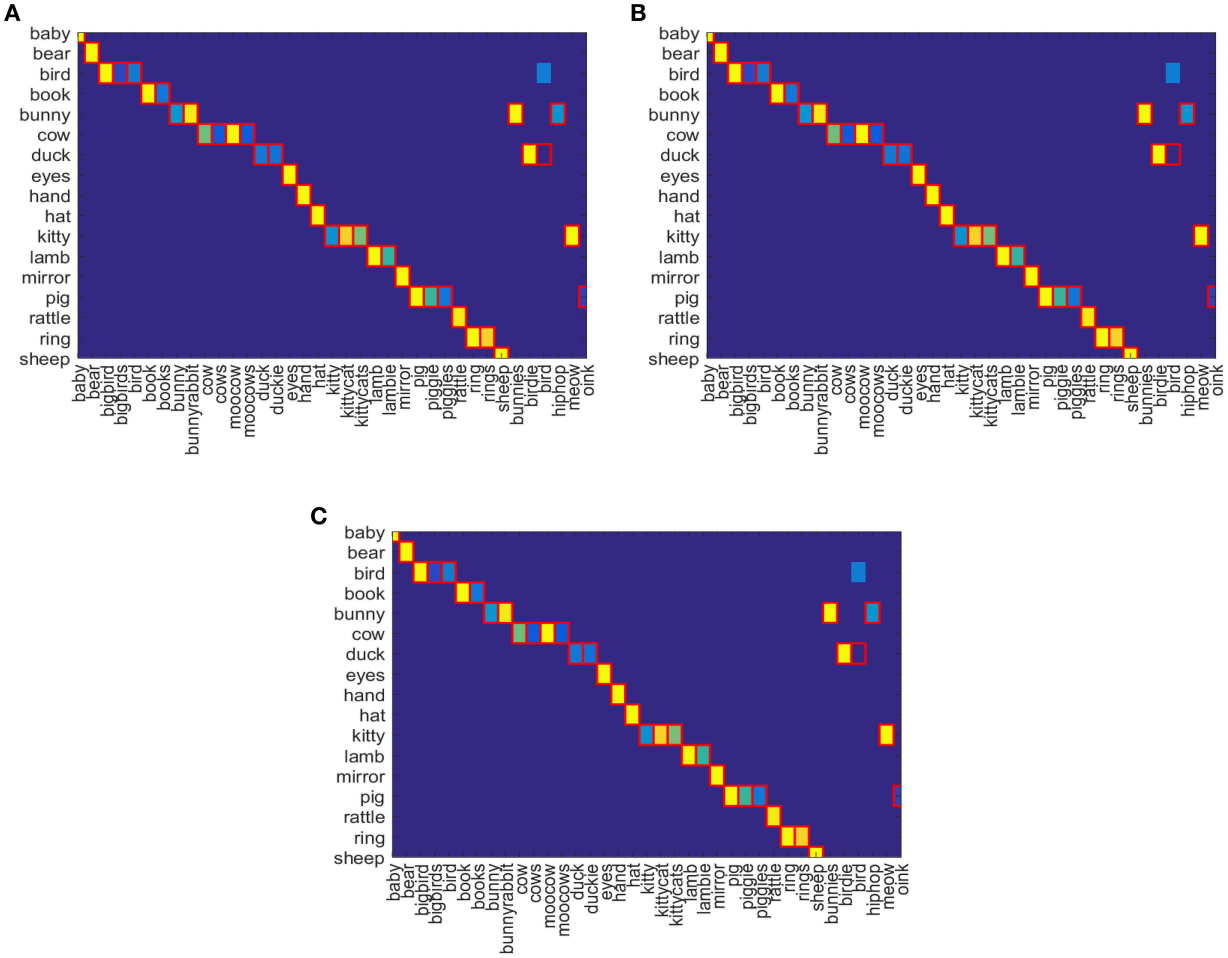
Confusion Matrix for **(A)** Attentional-prosodic Q-NN, **(B)** Attentional-prosodic NFQ, and **(C)** Attentional-prosodic DQN, respectively.

In Figures 7A–C, confusion matrix is depicted for gold standard word-object pairs using Equation (8) for Attentional-prosodic Q-NN, NFQ, and DQN. As before, the association between words and objects are shown as gradient of colors from yellow to blue. Figures 7A–C shows that Attentional-prosodic Q-NN, NFQ, and DQN correctly identify 36 word-object pairs out of 37 given target words. However, the algorithms chose “bird” to refer to <bird>only when it can also be associated with the object <duck>. So adding prosodic feature does not resolve the problem of discovering one word referring to multiple objects. This is expected because of the language constraints (“novel words to novel objects”) chosen in the proposed models for computing rewards.

**Figure 8:**
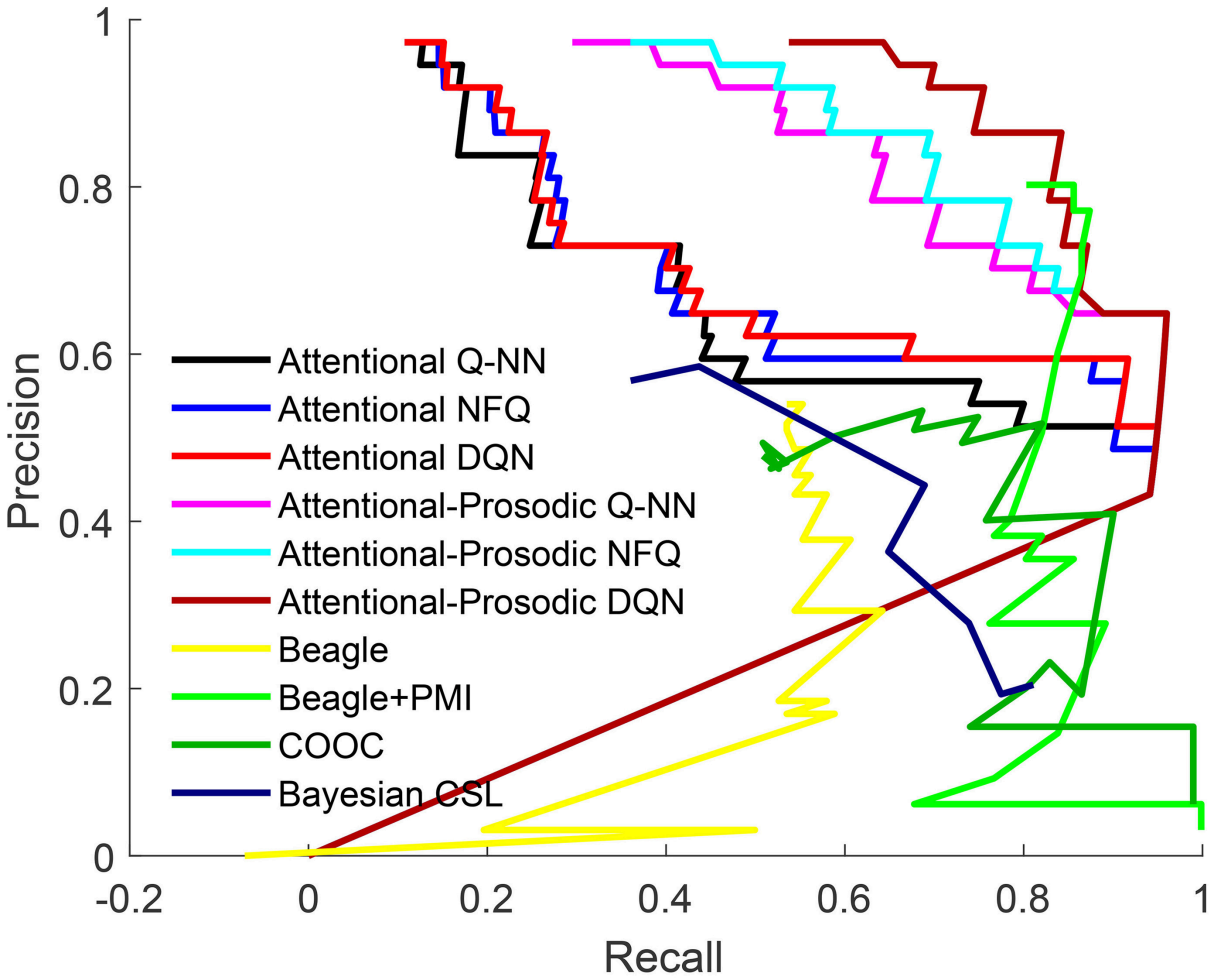
ROC for Attentional Q-NN, NFQ, DQN, Attentional-prosodic Q-NN, NFQ, DQN, existing Beagle, Beagle+PMI model, COOC, and Bayesian CSL.

To compare the performance of the proposed models with exiting models in the task of learning word meaning, we focus on precision, recall and F-score computed from each model. We have analyzed the effect of integrating prosodic cues with cross-situational learning in the proposed model and then compared it with the state-of-the-art models. To investigate the effect of addition of prosodic features, a lexicon is created from the *Q*-value matrix using a threshold, in the same way as was done for joint attention only. In order to compare the proposed models (Attentional Q-NN, NFQ, DQN, Attentional-prosodic Q-NN, NFQ, DQN) with existing ones (BEAGLE, hybrid BEAGLE+PMI, Bayesian CSL, COOC), the precision and recall for lexicons across the full range of threshold values is shown in Figure 8. Recall is higher for Attentional-prosodic models than Attentional models. Thus, integration of prosodic cues with joint attention helps the models to learn correct words among all the relevant words expected to be learnt.

The best F-score, precision and recall for Attentional Q-NN, NFQ, DQN, Attentional-prosodic Q-NN, NFQ, DQN, and existing Bayesian CSL, BEAGLE, hybrid (BEAGLE+PMI), MSG, and baseline COOC models are tabulated in Table 3. Attentional Q-NN and Attentional-prosodic Q-NN has highest precision of 95 and 96%, respectively. However, both have much lower recall than Attentional NFQ, DQN, Attentional-prosodic NFQ, DQN, and existing state-of-the-art Beagle+PMI model. On the other hand, fusion of prosody with joint attention in Attentional-prosodic NFQ and DQN enhances the models' capacity in terms of having better trade-off between precision and recall. Attentional-prosodic DQN has better F-score, precision and recall than Attentional-prosodic NFQ. Among all the reinforcement learning models considered in this paper, Attentional-prosodic DQN produces best performance for the task of word-learning.

**Table 3:** Comparison of proposed attentional and Attentional-prosodic reinforcement learning models with existing models.

	**F-score**	**Precision**	**Recall**
Attentional Q-NN	0.6667	**0.95**	0.5135
Attentional NFQ	0.7097	0.88	0.5946
Attentional DQN	0.7213	0.9167	0.5946
Attentional-prosodic Q-NN	0.77419	**0.96**	0.6486
Attentional-prosodic NFQ	0.78378	0.7838	0.7838
Attentional-prosodic DQN	**0.85333**	0.8421	**0.8641**
COOC	0.53	0.7578	0.4012
Bayesian CSL model	0.54	0.64	0.47
Beagle model	0.55	0.58	0.525
Beagle+prosodic cue model	0.6629	0.71	0.525
Beagle+PMI model	0.83	0.86	0.81
MSG model	0.64	NA	NA
Attentive MSG	0.7	NA	NA
AttentiveSocial MSG	0.73	NA	NA

*The bold values in each column indicate highest score over the algorithms*.

When compared to the existing models, Attentional DQN exhibits higher F-score than COOC, Bayesian CSL, BEAGLE, MSG, and AttentiveMSG models. It is noted that existing models mentioned here ignore prosodic cues. AttentiveMSG and Attentivesocial MSG models integrate social cues with cross-situational learning where infants attend to objects held by them instead of following eye gaze. State-of-the-art BEAGLE+PMI model also ignores prosodic cues in the infant-directed speech. For a fair comparison, we have tested BEAGLE model with prosodic vector instead of using random vectors. The BEAGLE model yields an F-score of 0.55 which increases to 0.6629 when it is integrated with prosodic cues. Integration of PMI model with prosodic cues is yet to be researched. No experiment with Attentional-prosodic BEAGLE+PMI model is performed in this paper due to availability of limited information regarding the exact procedure. Since the F-score of BEAGLE+PMI is very close to that of Attentional-prosodic DQN, it is unclear how the performance of the former would compare to reinforcement learning models. However, only joint attention could not make DQN's performance better than AttentiveSocial MSG. When prosody is combined with joint attention, DQN produces higher F-score than AttentiveSocialMSG and BEAGLE integrated with prosodic cue models. It is noteworthy that Attentional-prosodic DQN and BEAGLE+PMI would have been more comparable if the latter incorporated prosodic information as the former. The best lexicon learned by Attentional-prosodic DQN is shown in Table 4.

**Table 4:** Learned best lexicon (word-object pairs) using Attentional-prosodic DQN.

**Words**	**Objects**	**Words**	**Objects**	**Words**	**Objects**	**Words**	**Objects**
“ahhah”	eyes	“bunnies”	bunny	“hiphop”	bunny	“pig”	pig
“ahhah”	rattle	“bunny”	bunny	“david”	mirror	“piggie”	pig
“baby”	baby	“bunnyrabbit”	bunny	“kitty”	kitty	“piggies”	pig
“bear”	bear	“cow”	cow	“kittycat”	kitty	“rattle”	rattle
“big”	bunny	“duck”	duck	“kittycats”	kitty	“ring”	ring
“bigbird”	bird	“duckie”	duck	“lamb”	lamb	“rings”	ring
“bird”	bird	“eyes”	eyes	“lambie”	lamb	“sheep”	sheep
“birdie”	duck	“hand”	hand	“meow”	kitty	“through”	bunny
“book”	book	“hat”	hat	“mirror”	mirror		
“books”	book	“he”	duck	“moocow”	cow		

## 5. Discussion

In this paper, an agent is developed that can learn word-object pairings from ambiguous environments using reinforcement. Joint attention and prosodic cues are integrated in caregiver's speech with cross-situational learning. Prosodic cues are extracted from audio stream. Joint attention is utilized to compute the reward for the agent. Among the proposed Q-NN, NFQ, and DQN algorithms for word-learning, Q-NN is online whereas the other two use batch processing. According to the behavioral studies in Vouloumanos (2008) and Yu and Smith (2007), the human brain follows an incremental learning algorithm for the task of word-learning. Moreover, it is claimed that only memory-limited models can truly mimic human performance (Frank et al., 2010). In Medina et al. (2011), it is found that cross-situational word-learning is sensitive to input order which is incompatible with the prediction of ideal learners assuming full access to statistical regularities in data. Among neural network-based word-learning models used in this paper, Q-NN requires the memory that contains the information only from the last trial which can be called memory-limited. In case of NFQ and DQN, both models have an episodic memory for storing transition data samples and corresponding rewards of the most recent 50 experiences. In Q-NN, learning update rule is online/incremental and follows the input order of the dataset. On the other hand, for NFQ the update is performed through batch processing of the batch size of the storage. In DQN, each time a mini-batch of size 10 is randomly selected from episodic memory to update the neural network parameters. So, Q-NN has higher fidelity to human performance than the other two models, NFQ and DQN, though it provides lower F-score than NFQ and DQN.

Some research (e.g., Yu, 2008; Kachergis et al., 2012a; Vlach and Sandhofer, 2012) suggests that episodic memory, a basic human cognitive ability, likely supports language-learning. According to this hypothesis, objects are stored in memory along with words and other stimuli we encounter in every situation (i.e., in a language-learning class or while eating breakfast). If the word-object pair is confirmed by being consistent with the succeeding observation, the learner will further solidify the word meaning in memory (Medina et al., 2011; Trueswell et al., 2013). According to this hypothesis, episodic memory can play an important role in word-referent pair learning. This hypothesis is supported by our proposed models, NFQ and DQN, where both have storage capability. However, from a computational perspective, DQN provides the best F-score compared to other models.

It is found through behavioral studies in Estes and Bowen (2013) that the increased pitch and variation in pitch contours of infant-directed speech attract attention and enhance arousal for word-learning and infants in the infant-directed speech condition were able to learn the associations successfully. Another study (Zangl and Mills, 2007) proposed that prosodic characteristics in infant-directed speech allows infants to form stronger associations between words and their referents because of increase in brain activation for prosodic words. In these behavioral prosodic studies, joint attention is ignored. In Filippi et al. (2014), behavioral studies are performed to compare the learning effects of typical pitch emphasis in infant-directed speech with those of other visual and acoustic attentional cues. It is found that word-object pair learning performance is better when consistent high pitch of the target word is integrated with co-occurrence as compared to co-occurrence alone or co-occurrence integrated with visual cues. Simulation results using a mother-infant interaction dataset show that, for given gold-standard words, both Attentional Q-NN, NFQ, DQN, and Attentional-prosodic Q-NN, NFQ, DQN models can learn correct associations with 97.29% accuracy (36 out of 37). When prosodic cues are included along with joint attention, they learn higher number of correct word-object pairs in comparison to using only joint attention, which is consistent with experimental studies (Filippi et al., 2014) and computational models (Yu and Ballard, 2007).

From a machine learning perspective, the proposed Attentional-prosodic DQN model outperforms some of the existing models in word-learning tasks in terms of F-score, precision and recall. However, our models failed to discover the association if one word is assigned to multiple objects due to assuming the “novel words to novel objects” language-specific constraint for computing rewards. A future goal is to model reinforcement by relaxing the language constraint. It is believed that the word-learning process in children is incremental. Though the proposed Attentional-prosodic Q-NN model-based agent learned incrementally, our best performing agent is based on Attentional-prosodic DQN model which learns in mini-batches. In the proposed approach, joint attention was manually selected from the video. Recently a number of neural network-based reinforcement learning models have manifested strong performance in computing visual joint attention (Doniec et al., 2006; da Silva and Romero, 2011; Da Silva and Romero, 2012). In future, it would be interesting to integrate automatic detection of joint attention with the proposed approach to make the proposed word-learning model more general.

## Author contributions

SN: Conducted the research on the algorithms, implemented and evaluated them, and wrote the paper; BB: Played an advisory role, helped in framing the problem and writing the paper, and acquired funding for conducting this research.

### Conflict of interest statement

The authors declare that the research was conducted in the absence of any commercial or financial relationships that could be construed as a potential conflict of interest.
